# Growth, Specialty Distribution, and Regulatory Pathways of Artificial Intelligence-Enabled Medical Devices Authorized in the United States From 1995 to 2026: A Cross-Sectional Analysis

**DOI:** 10.7759/cureus.119039

**Published:** 2026-11-03

**Authors:** Suhail S Alshahrani

**Affiliations:** 1 Department of Biomedical Technology, College of Applied Medical Sciences, King Saud University, Riyadh, SAU

**Keywords:** 510(k) clearance, artificial intelligence, de novo classification, food and drug administration, machine learning, medical device regulation, medical imaging, post-market surveillance, regulatory science

## Abstract

Introduction

The United States Food and Drug Administration (FDA) publishes a list of artificial intelligence (AI)-enabled medical devices authorized for marketing. Annual authorizations have risen sharply in recent years, and an updated analysis of the complete list is needed. This study aimed to characterize regulatory authorizations on the full list through mid-2026.

Methods

A cross-sectional analysis of all 1,614 entries on the FDA AI-Enabled Medical Device List (final decisions from September 1995 to June 2026) was performed. Regulatory pathway, year, lead review panel, product code, and manufacturer group were derived for each entry. Growth was estimated by log-linear regression, trends were tested with the Cochran-Armitage test and logistic regression with manufacturer-clustered standard errors, and associations were tested with chi-squared and Fisher exact tests, with Holm adjustment for multiple testing.

Results

Annual authorizations rose from 18 in 2016 to 335 in 2025, an estimated growth of 35.3% per year (95% confidence interval (CI) 27.0-44.3), and 977 of 1,614 entries (60.5%) were authorized from 2023 onward. Radiology accounted for 1,230 entries (76.2%); its share peaked at 347 of 405 (85.7%) in 2020-2022 and fell to 750 of 977 (76.8%) in 2023-2026 (p < 0.001), while radiology authorizations kept growing in absolute terms and the number of represented specialty panels rose from 10 to 16. The 510(k) pathway accounted for 1,553 authorizations (96.2%), and radiology entries were less likely than others to use the De Novo or premarket approval pathways (18 of 1,230, 1.5% vs. 43 of 384, 11.2%; odds ratio 0.14, 95% CI 0.08-0.27 after adjustment for era). Of 40 De Novo grants, 22 created product codes that later received 181 510(k) clearances. Imaging systems such as ultrasound, computed tomography, and magnetic resonance scanners accounted for 399 entries (24.7%), and seven large manufacturers accounted for 288 of these (72.2%). Findings were similar for 1,195 deduplicated devices and for entries through December 2025.

Conclusions

FDA authorization of AI-enabled devices continues to accelerate. Radiology remains dominant and still growing, while other specialties are expanding alongside it; the 510(k) pathway predominates, and a growing share of authorizations concerns AI embedded in imaging equipment. These findings describe regulatory authorizations rather than clinical adoption, and they identify transparency about AI functions in conventional devices and AI-specific post-market surveillance as topics for further study.

## Introduction

Artificial intelligence (AI), particularly machine learning (ML), has moved from research prototypes into authorized medical devices [1,2]. Authorized applications include detection and triage of findings on medical images, image reconstruction and noise reduction in scanners, arrhythmia detection in cardiac monitors, and risk prediction from physiological signals [2,3]. Because these functions can influence clinical decisions, AI-enabled software that meets the definition of a medical device is subject to premarket review by regulators such as the United States Food and Drug Administration (FDA) [4].

In the United States, devices reach the market through one of three main routes. Premarket notification, or 510(k), requires demonstrating substantial equivalence to a legally marketed predicate device. The De Novo pathway classifies novel low-to-moderate-risk devices that have no predicate and creates a new classification regulation and product code. Premarket approval (PMA) is required for high-risk devices and demands evidence of safety and effectiveness [5]. The De Novo pathway was introduced in 1997, and since 2012, sponsors have been able to request De Novo classification directly, without first receiving a not-substantially-equivalent decision on a 510(k) submission [5]. The pathway therefore reflects both novelty and evidentiary burden, and later devices can use earlier ones, including those authorized through De Novo, as predicates.

To improve transparency, the FDA maintains a public list of AI-enabled medical devices authorized for marketing in the United States [4]. The list identifies devices mainly by searching decision summaries and device classifications for AI-related terms drawn from the FDA Digital Health and Artificial Intelligence Glossary, and the agency notes that it is not exhaustive [4,6]. Several studies have used this list or similar sources to describe the field. Benjamens et al. compiled an early database of AI-based FDA authorizations [1]. Muehlematter et al. compared FDA and European approvals for 2015-2020 [7] and later showed that many AI devices cleared through 510(k) trace their predicate networks back to devices without AI functionality [8]. Wu et al. highlighted the predominance of retrospective, single-site evaluations [9], Lyell et al. examined how ML functions support clinical decisions [3], Zhu et al. described the 2021 landscape [10], and Joshi et al. analyzed 691 devices listed by October 2023, of which 531 (76.8%) were radiology devices, and nearly all were cleared through 510(k) [2]. Two recent analyses extended this work to December 2025: Lee and Youn examined clinical specialty expansion across four prespecified eras using multivariable models with manufacturer-clustered standard errors [11], and Golshani and Joseph described persistent specialty concentration and a fragmented manufacturer base [12].

The regulatory framework has also evolved, with FDA final guidance on predetermined change control plans for AI-enabled device software functions, issued in December 2024 and updated in August 2025 [13], and draft guidance on lifecycle management and marketing submissions in January 2025 [14], building on good ML practice principles issued jointly with Health Canada and the United Kingdom Medicines and Healthcare products Regulatory Agency [15]. Other regulators have issued their own frameworks, including the Saudi Food and Drug Authority (SFDA) guidance MDS-G010 [16,17] and the European Union AI Act [18]. Because the FDA list is one of the few public registries of authorized AI-enabled devices and is widely used in international comparisons [7,11], an up-to-date picture of FDA practice is useful beyond the United States.

Recent analyses have focused mainly on specialty and manufacturer distributions [11,12]. Beyond extending the observation period by six months, this study makes three distinct contributions. First, it compares regulatory pathway use across review panels and tests whether differences persist after accounting for era. Second, it analyzes product codes, including how De Novo classifications are reused by later 510(k) clearances. Third, it quantifies authorizations for conventional imaging systems, such as ultrasound, computed tomography (CT), and magnetic resonance (MR) scanners, that incorporate AI functions. This last distinction may matter to clinicians and health systems, because the AI component of such a device may be less visible to users than standalone software.

This study aimed to provide an updated analysis of regulatory authorizations on the FDA AI-Enabled Medical Device List through June 2026. Because the list records marketing authorizations, the study describes regulatory activity rather than clinical adoption, use, or outcomes [11]. The specific objectives were to describe the growth of authorizations over time and their distribution across clinical specialties; to examine regulatory pathway use by specialty, including whether differences persist after accounting for era, and the role of De Novo grants in establishing product codes later used by 510(k) clearances; to quantify authorizations made under imaging system product codes; and to describe the structure of the manufacturer base.

## Materials and methods

Study design and data source

This cross-sectional, descriptive analysis used publicly available regulatory data. The data source was the FDA AI-Enabled Medical Devices List [4], as of October 15, 2026 (page last updated September 4, 2026). The complete table was copied from the FDA web page on that date because the downloadable CSV and Excel files were not accessible at the time, and it contained 1,614 entries with six fields: date of final decision, submission number, device name, company, lead review panel, and primary product code. Each entry corresponds to one marketing authorization with a unique submission number. Successive versions of the same product authorized under separate submissions therefore appear as separate entries. The unit of analysis is the list entry, that is, one marketing authorization; the terms entry and authorization are used interchangeably, and the term device is used only for the deduplicated sensitivity analysis described below or for devices in general. Final decision dates ranged from September 29, 1995, to June 29, 2026. Because the study used only public regulatory records and involved no human participants or personal data, it did not require ethics committee approval.

Data processing

One entry split across two lines during copying was merged, after which all entries had complete values in all six fields, and no duplicate submission numbers were present. The review panel label "Clinical Toxcicology" in the source was corrected to "Clinical Toxicology." No values were missing in the source fields or in any derived variable (pathway, period, specialty group, imaging system flag, and manufacturer group), so no entries were excluded, and no imputation was needed. The following variables were derived.

Regulatory Pathway

The pathway was assigned from the submission number prefix: K for 510(k), DEN for De Novo, and P for PMA, including PMA supplements identified by the suffix "/S". Every submission number carried one of these three prefixes.

Time

The calendar year was taken from the final decision date. Four periods were defined in advance to correspond to recognized phases of AI device development and regulation: 1995-2015, before the widespread use of deep learning in medical devices; 2016-2019, the early deep learning period; 2020-2022, a period of rapid digital health expansion that coincided with the COVID-19 pandemic; and January 2023 to June 2026, marked by the emergence of foundation models and the FDA predetermined change control plan framework [13]. These boundaries are the same as the eras defined independently by Lee and Youn on the basis of authorization trends and technological and regulatory milestones [11]. Because the periods differ in length (21, four, three, and 3.5 years), comparisons between periods are based on proportions within each period, and authorization volume is also expressed as an annualized rate. The final period includes only the first six months of 2026, which were excluded from growth models and are shown separately in figures.

Specialty

The lead review panel was used as a proxy for clinical specialty. For some analyses, panels were grouped into radiology, cardiovascular, neurology, and all other panels.

Imaging Systems

Entries whose primary product code belongs to a classification regulation for imaging acquisition equipment were flagged as imaging system entries. The codes were IYN and IYO (diagnostic ultrasound), JAK (CT), LNH (MR), KPS (emission CT), OUO (combined positron emission tomography and MR), OWB, OXO, and JAA (fluoroscopic X-ray), KPR, MQB, and IZL (radiographic X-ray), and OTE (digital breast tomosynthesis); the mapping with the number of entries per code is given in Appendix A. This classification is based on the regulatory product code and not on a review of each device, which is discussed as a limitation.

Manufacturers

Company names were standardized by converting them to lowercase and removing punctuation, parenthetical text, trade-name qualifiers, and legal suffixes (for example, Inc., Ltd., LLC, GmbH, and B.V.). Names belonging to the same corporate group were then merged using a rule list, so that, for example, GE Medical Systems, GE Healthcare Japan, and GE Hangwei Medical Systems were grouped as GE HealthCare. The full rule list is given in Appendix B. Seven firms with large imaging equipment portfolios (GE HealthCare, Siemens Healthineers, Philips, Canon, United Imaging, Fujifilm, and Samsung) were defined in advance as major imaging manufacturers.

De Novo Follow-On Clearances

For each De Novo grant, the number of later 510(k) clearances under the same primary product code was counted, as an indicator of how often a De Novo classification became the basis for subsequent substantial equivalence submissions.

Unique Devices

For a sensitivity analysis, entries were grouped into unique devices by manufacturer group and a normalized device name, obtained by keeping the first listed product name; removing version numbers, parenthetical text, and trademark symbols; and removing generic words such as software, system, and platform. This produced 1,195 unique devices from the 1,614 entries.

Statistical analysis

Categorical variables are reported as counts and percentages, with Wilson 95% confidence intervals (CIs) for the main proportions. Growth in annual authorizations between 2016 and 2025 was estimated by ordinary least squares regression of the natural logarithm of annual counts on calendar year, and the slope was converted to an annual percentage change with a 95% CI; the compound annual growth rate over the same interval is also reported. The 2016 start corresponds to the beginning of the early deep learning period; because this is also where the steepest growth begins, the model was repeated from 2010 as a quasi-Poisson regression of annual counts on calendar year for 2010-2025. Linear trends in proportions across the four periods were tested with the Cochran-Armitage test, using period scores of zero, one, two, and three. Because this test assumes a monotonic trend and equally spaced scores, changes in the radiology share were also modeled with logistic regression on decimal year from 2016 onward, with a change in slope at January 1, 2023. Proportions in two periods were compared with the Pearson chi-squared test, and pathway use across specialty groups was compared with the chi-squared test of independence. The association between radiology and use of a novel pathway (De Novo or PMA) was summarized with the two-sided Fisher exact test and an unadjusted odds ratio (OR), and then with logistic regression adjusted for period or for decimal year, and in the subset from 2012 onward, when direct De Novo requests became available. Because entries from the same manufacturer may be correlated, all logistic models used cluster-robust standard errors clustered by manufacturer group (559 clusters). Specialty diversity in each period was summarized with the Shannon diversity index across review panels, and market concentration with the Herfindahl-Hirschman Index (HHI), computed as the sum of squared percentage shares of manufacturer groups. The seven hypothesis tests reported in the Results section (three Cochran-Armitage tests, three chi-squared tests, and one Fisher exact test) were treated as one family and adjusted with the Holm method to control the family-wise error rate at 0.05, and adjusted p-values are reported for them. Panels with fewer than 30 entries are described without inferential comparison. Sensitivity analyses repeated the main estimates for unique devices and for entries through December 2025. Analyses were performed in Python 3 (Python Software Foundation, Wilmington, DE, USA) using the pandas, NumPy, and SciPy libraries, with logistic models fitted by maximum likelihood and sandwich variance estimators, and figures were produced with Matplotlib.

## Results

Overview

The list included 1,614 authorizations of AI-enabled devices between 1995 and June 2026, submitted under 816 distinct company names that standardized to 559 manufacturer groups. Most were cleared through 510(k) (1,553; 96.2%), followed by De Novo (40; 2.5%) and PMA (21; 1.3%), including four PMA supplements. Entries were spread across 18 lead review panels and 181 primary product codes. Table 1 summarizes the list's characteristics by period.

**Table 1:** Characteristics of FDA AI-Enabled Medical Device List entries (authorizations) by period of final decision. Values are n (%) with Wilson 95% confidence intervals (CIs) in parentheses where shown. Annualized rates divide the number of authorizations by the period length (21, four, three, and 3.5 years). Seven major imaging manufacturers: GE HealthCare, Siemens Healthineers, Philips, Canon, United Imaging, Fujifilm, and Samsung; their row counts authorizations across all product codes. FDA: United States Food and Drug Administration; PMA: premarket approval

Characteristic	1995-2015	2016-2019	2020-2022	2023-2026 (January 2023 to June 2026)	Total
Authorizations, n (% of all entries)	42 (2.6)	190 (11.8)	405 (25.1)	977 (60.5)	1,614 (100.0)
Annualized authorizations per year	2.0	47.5	135.0	279.1	Not applicable
Radiology panel, n (%) (95% CI)	15 (35.7) (23.0-50.8)	118 (62.1) (55.0-68.7)	347 (85.7) (81.9-88.8)	750 (76.8) (74.0-79.3)	1,230 (76.2) (74.1-78.2)
Cardiovascular panel, n (%) (95% CI)	4 (9.5) (3.8-22.1)	36 (18.9) (14.0-25.1)	33 (8.1) (5.9-11.2)	81 (8.3) (6.7-10.2)	154 (9.5) (8.2-11.1)
Neurology panel, n (%) (95% CI)	2 (4.8) (1.3-15.8)	12 (6.3) (3.6-10.7)	6 (1.5) (0.7-3.2)	53 (5.4) (4.2-7.0)	73 (4.5) (3.6-5.6)
Review panels represented, n	11	15	10	16	18
Shannon diversity index (panels)	2.04	1.34	0.62	1.00	Not applicable
De Novo or PMA, n (%) (95% CI)	11 (26.2) (15.3-41.1)	12 (6.3) (3.6-10.7)	10 (2.5) (1.3-4.5)	28 (2.9) (2.0-4.1)	61 (3.8) (3.0-4.8)
Imaging system product codes, n (%) (95% CI)	4 (9.5) (3.8-22.1)	27 (14.2) (10.0-19.9)	120 (29.6) (25.4-34.3)	248 (25.4) (22.8-28.2)	399 (24.7) (22.7-26.9)
Authorizations held by seven major imaging manufacturers (all product codes), n (%) (95% CI)	2 (4.8) (1.3-15.8)	18 (9.5) (6.1-14.5)	123 (30.4) (26.1-35.0)	236 (24.2) (21.6-26.9)	379 (23.5) (21.5-25.6)

Growth of authorizations

Only 42 entries on the list were authorized before 2016. Annual authorizations then rose from 18 in 2016 to 114 in 2020 and 335 in 2025 (Figure 1). The log-linear model estimated an average growth of 35.3% per year between 2016 and 2025 (95% CI 27.0-44.3; R² = 0.937), and the compound annual growth rate over the same interval was 38.4%. Growth estimates were similar when the model started in 2010 (44.5% per year, 95% CI 38.7-50.6) and in a quasi-Poisson model for 2010-2025 (34.8% per year, 95% CI 29.9-39.9). Expressed as annualized rates, the four periods averaged 2.0, 47.5, 135.0, and 279.1 authorizations per year. In 2025, authorizations reached a record 335, up from 235 in 2024 (a 42.6% increase). The first half of 2026 included 181 authorizations, compared with 176 in the first half of 2025, corresponding to an annualized rate of 362 against the 335 recorded in 2025; because of reporting lag, the 2026 figure is provisional. The cumulative number of entries passed 100 in 2018, 500 in 2022, and 1,000 in 2024. Overall, 1,382 of 1,614 entries (85.6%) were authorized from 2020 onward and 977 (60.5%) from 2023 onward.

**Figure 1:**
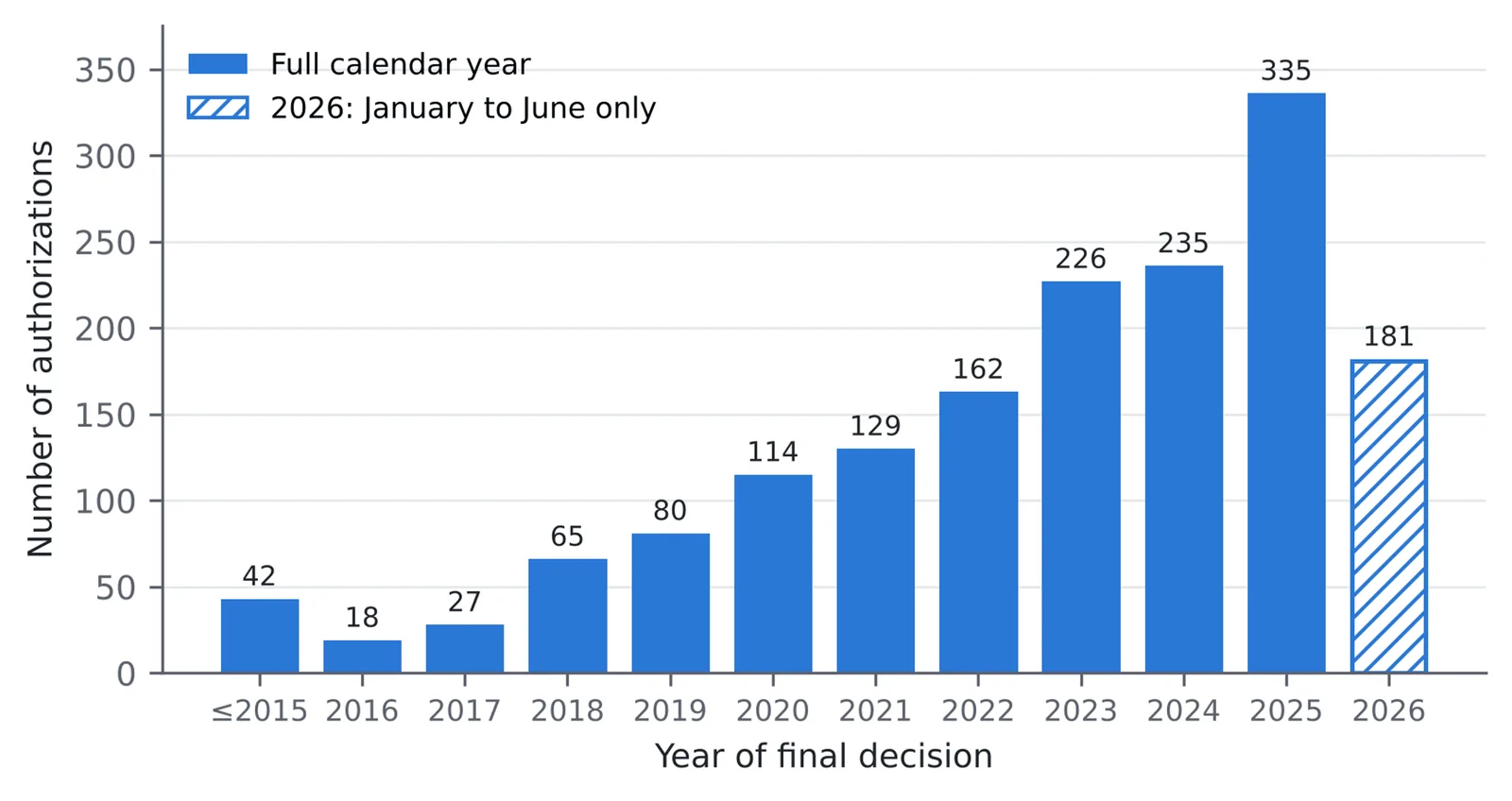
Annual number of authorizations on the FDA AI-Enabled Medical Device List. Bar labels show the number of entries (authorizations). Entries from 1995 to 2015 are pooled. The hatched bar for 2026 covers January to June only. FDA: United States Food and Drug Administration

Specialty distribution

Radiology was the lead review panel for 1,230 entries (76.2%; 95% CI 74.1-78.2), followed by cardiovascular (154; 9.5%), neurology (73; 4.5%), anesthesiology (30; 1.9%), gastroenterology-urology (27; 1.7%), and hematology (22; 1.4%). The remaining 12 panels together accounted for 78 entries (4.8%) and are described without inferential comparison because of their small numbers (Table 2).

**Table 2:** Distribution of list entries (authorizations) by lead review panel and regulatory pathway. Panels with fewer than 30 entries are described without inferential comparison. PMA: premarket approval (including PMA supplements)

Lead review panel	510(k), n	De Novo, n	PMA, n	Total, n (%)
Radiology	1,212	9	9	1,230 (76.2)
Cardiovascular	144	6	4	154 (9.5)
Neurology	69	4	0	73 (4.5)
Anesthesiology	26	4	0	30 (1.9)
Gastroenterology-urology	25	2	0	27 (1.7)
Hematology	22	0	0	22 (1.4)
Pathology	4	3	3	10 (0.6)
Ophthalmic	8	2	0	10 (0.6)
General and plastic surgery	3	2	4	9 (0.6)
Clinical chemistry	7	2	0	9 (0.6)
Orthopedic	8	0	0	8 (0.5)
Dental	5	2	0	7 (0.4)
Microbiology	6	1	0	7 (0.4)
General hospital	5	1	0	6 (0.4)
Obstetrics and gynecology	4	0	1	5 (0.3)
Clinical toxicology	5	0	0	5 (0.3)
Ear, nose, & throat	0	1	0	1 (0.1)
Immunology	0	1	0	1 (0.1)
Total	1,553	40	21	1,614 (100.0)

The specialty mix changed over time in a non-monotonic way (Table 1, Figure 2). In the early period, radiology accounted for only 15 of 42 entries (35.7%; 95% CI 23.0-50.8), with hematology, clinical toxicology, and other laboratory devices making up much of the remainder. The radiology share rose to 118 of 190 (62.1%; 95% CI 55.0-68.7) in 2016-2019, peaked at 347 of 405 (85.7%; 95% CI 81.9-88.8) in 2020-2022, and was 750 of 977 (76.8%; 95% CI 74.0-79.3) in 2023-2026. The Cochran-Armitage test across the four periods was significant (z = 4.85, Holm-adjusted p < 0.001), but because the pattern rises and then falls, this result mainly reflects the earlier increase. In the logistic model from 2016 onward, the odds of an entry being a radiology entry increased by 22.8% per year up to the end of 2022 (OR 1.23 per year, 95% CI 1.10-1.37) and decreased by 18.1% per year from 2023 (OR 0.82, 95% CI 0.72-0.93). The 2023-2026 share was lower than the 2020-2022 share (χ² = 13.9, Holm-adjusted p < 0.001; manufacturer-clustered OR 0.55, 95% CI 0.39-0.78) but similar to the pooled share before 2023 (480 of 637, 75.4%), so the change is best described as a reversal from the 2020-2022 peak rather than a decline below earlier levels. Radiology authorizations continued to grow in absolute terms, from 347 in 2020-2022 to 750 in 2023-2026, and numbered 254 in 2025 alone. In parallel, the number of panels represented rose from 10 to 16, and the Shannon diversity index rose from 0.62 to 1.00.

**Figure 2:**
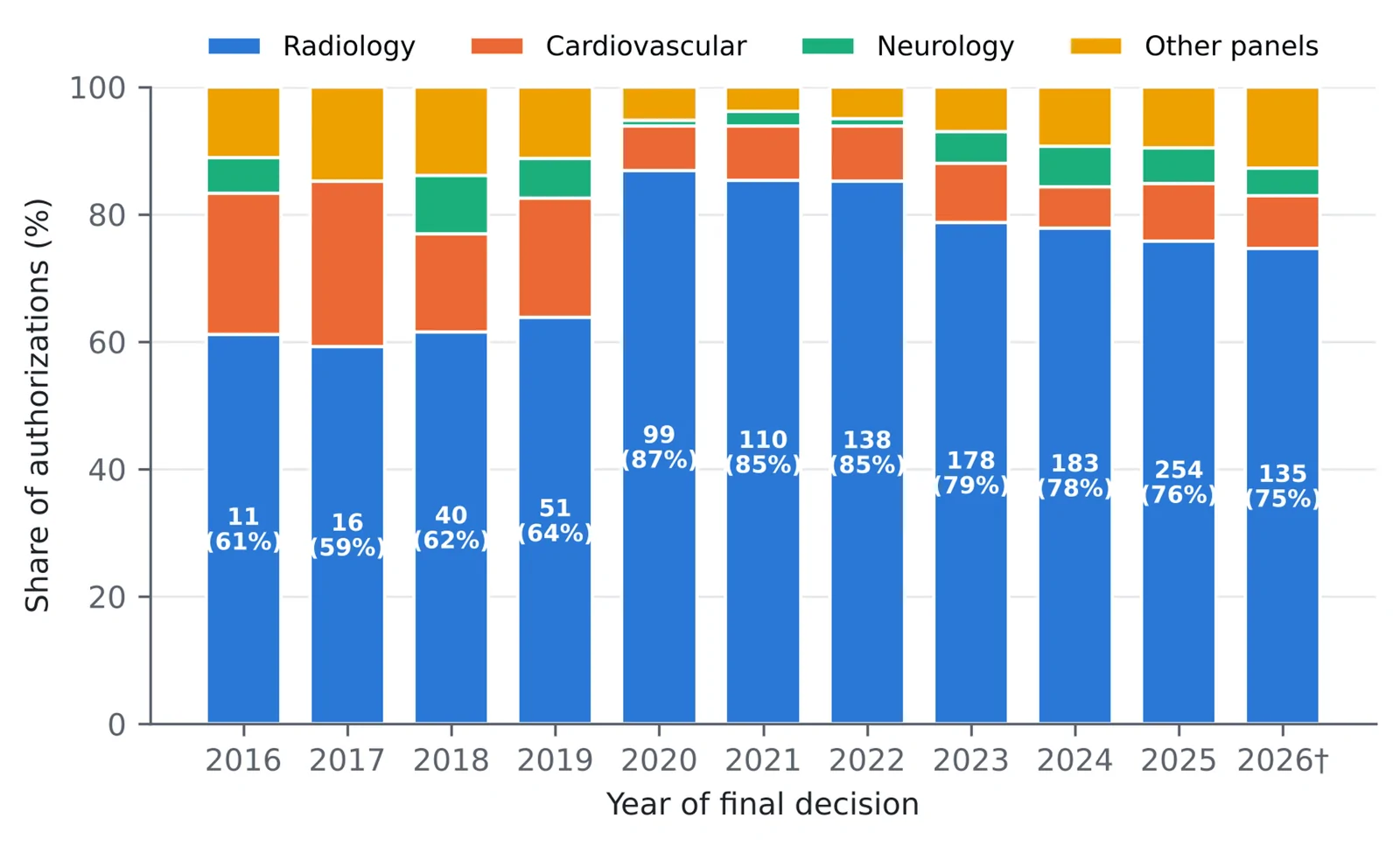
Share of annual authorizations by lead review panel, 2016-2026. Labels show the number and percentage of radiology entries in each year. The 2026 bar (marked with a dagger) covers January to June only.

Between 2020-2022 and 2023-2026, neurology entries rose from six to 53, mostly surgical navigation and robotic guidance systems (21 entries) and EEG and sleep analysis software (14 entries); anesthesiology entries rose from three to 22, mostly home sleep apnea testing (11) and lung sound analysis (four); and gastroenterology-urology entries rose from four to 22, of which 16 were computer-aided polyp detection systems. Cardiovascular entries rose from 33 to 81, led by ECG analysis and electrophysiology mapping software (20). These counts are small and are presented descriptively.

Regulatory pathways

The proportion of entries authorized through a novel pathway (De Novo or PMA) declined from 11 of 42 (26.2%) in the early period to 12 of 190 (6.3%) in 2016-2019, 10 of 405 (2.5%) in 2020-2022, and 28 of 977 (2.9%) in 2023-2026 (Cochran-Armitage z = -5.32, Holm-adjusted p < 0.001). Pathway use differed by specialty (χ² = 115.03, df = 3, Holm-adjusted p < 0.001) (Figure 3).

**Figure 3:**
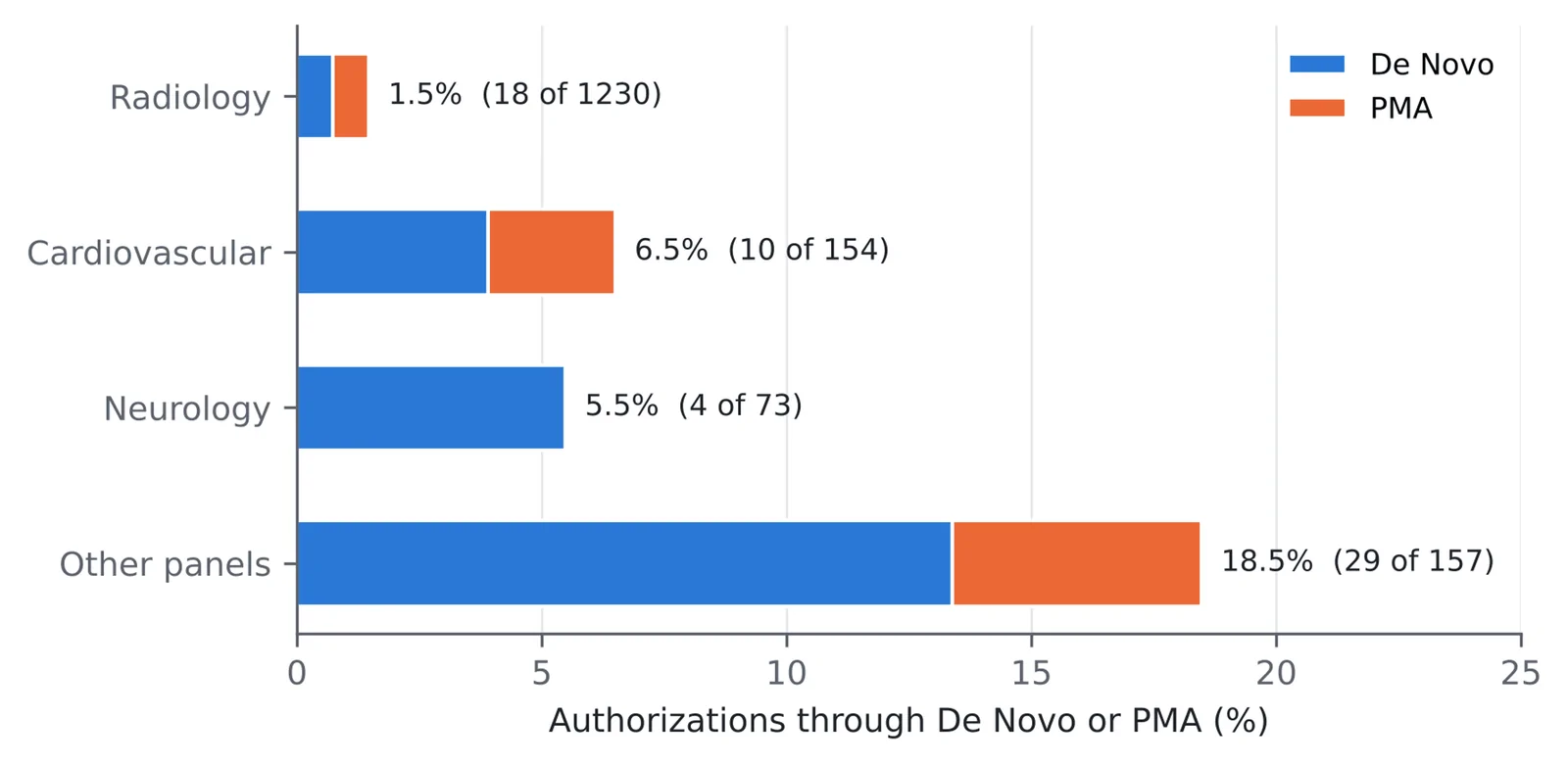
Proportion of entries authorized through the De Novo or premarket approval (PMA) pathways by review panel group. Bar labels show the percentage and the number of entries (n of total). All remaining entries were cleared through 510(k).

Novel pathways accounted for 18 of 1,230 radiology entries (1.5%; 95% CI 0.9-2.3), 10 of 154 cardiovascular entries (6.5%; 95% CI 3.6-11.5), four of 73 neurology entries (5.5%; 95% CI 2.2-13.3), and 29 of 157 entries from all other panels (18.5%; 95% CI 13.2-25.3). Compared with all non-radiology entries (43 of 384, 11.2%), radiology entries had lower odds of a De Novo or PMA authorization (unadjusted OR 0.12, 95% CI 0.07-0.21; Fisher exact Holm-adjusted p < 0.001). The association persisted after adjustment for period (OR 0.14, 95% CI 0.08-0.27) and for decimal year (OR 0.14, 95% CI 0.08-0.25), and in the subset from 2012 onward (OR 0.10, 95% CI 0.06-0.19), with manufacturer-clustered standard errors (Table 3).

**Table 3:** Odds of authorization through the De Novo or premarket approval pathways for radiology compared with all other review panels. Odds ratios compare radiology with all other panels. All logistic models used cluster-robust standard errors clustered by manufacturer group. The period model uses 2023-2026 as the reference period. CI: confidence interval

Model	Entries, n	Odds ratio (95% CI)	p-value
Unadjusted	1,614	0.12 (0.07-0.21)	p < 0.001
Adjusted for period (four periods)	1,614	0.14 (0.08-0.27)	p < 0.001
Adjusted for decimal year	1,614	0.14 (0.08-0.25)	p < 0.001
Entries from 2012 onward, adjusted for decimal year	1,593	0.10 (0.06-0.19)	p < 0.001
Entries through December 2025, adjusted for decimal year	1,433	0.15 (0.08-0.27)	p < 0.001
Unique devices, unadjusted (Fisher exact)	1,195	0.13 (not estimated)	p < 0.001

Each of the 40 De Novo grants on the list was associated with a distinct primary product code. Of these, 22 product codes subsequently received at least one 510(k) clearance, totaling 181 follow-on clearances, which is 11.7% of the 1,553 510(k) clearances on the list. Of the 19 De Novo grants made before 2023, 16 had already been followed by 510(k) clearances under the same code. The largest example was a 2018 De Novo grant for software that notifies stroke teams of suspected large vessel occlusion on CT angiography, which established the radiological computer-assisted triage and notification product code QAS; this code was subsequently used by 85 further 510(k) clearances from 27 manufacturer groups (Table 4).

**Table 4:** De Novo grants followed by the largest numbers of 510(k) clearances under the same product code. Later 510(k) clearances include submissions from the original sponsor and from other manufacturers. Device functions are described in the device listings. CT: computed tomography; MR: magnetic resonance

Device function (De Novo grant)	Submission number	Year of grant	Product code	Later 510(k) clearances, n
Notification of suspected large vessel occlusion on CT angiography	DEN170073	2018	QAS	85
Computer-aided diagnosis for breast MR imaging	DEN170022	2017	POK	21
Computer-aided polyp detection during colonoscopy	DEN200055	2021	QNP	19
Detection of wrist fractures on radiographs	DEN180005	2018	QBS	9
Guidance for acquisition of cardiac ultrasound images	DEN190040	2020	QJU	7
Contactless camera-based measurement of vital signs	DEN200019	2021	QME	6

Product codes and AI embedded in imaging systems

Product code use was highly concentrated. The five most frequent codes accounted for 793 entries (49.1%) and the 10 most frequent for 1,044 (64.7%), whereas 87 of the 181 codes appeared only once. The most frequent codes were QIH, automated radiological image processing software (305); LLZ, radiological image processing systems (161); IYN, diagnostic ultrasound systems (124); JAK, CT systems (103); LNH, MR systems (100); and QAS, radiological triage and notification software (86). New product codes continued to appear: 80 codes first appeared in 2023-2026, compared with 23 in 2020-2022.

Entries under imaging system product codes numbered 399 of 1,614 (24.7%), mainly ultrasound (136), CT (103), and MR (100) systems, followed by emission tomography (26) and fluoroscopic and radiographic X-ray systems. Their share of all authorizations rose from four of 42 (9.5%) in the early period and 27 of 190 (14.2%) in 2016-2019 to 120 of 405 (29.6%) in 2020-2022 (Cochran-Armitage z = 2.83, Holm-adjusted p = 0.009 across all periods) and remained at a similar level in 2023-2026 (248 of 977, 25.4%; Holm-adjusted p = 0.104 versus 2020-2022) (Figure 4). The seven major imaging manufacturers held 288 of these 399 listings (72.2%).

**Figure 4:**
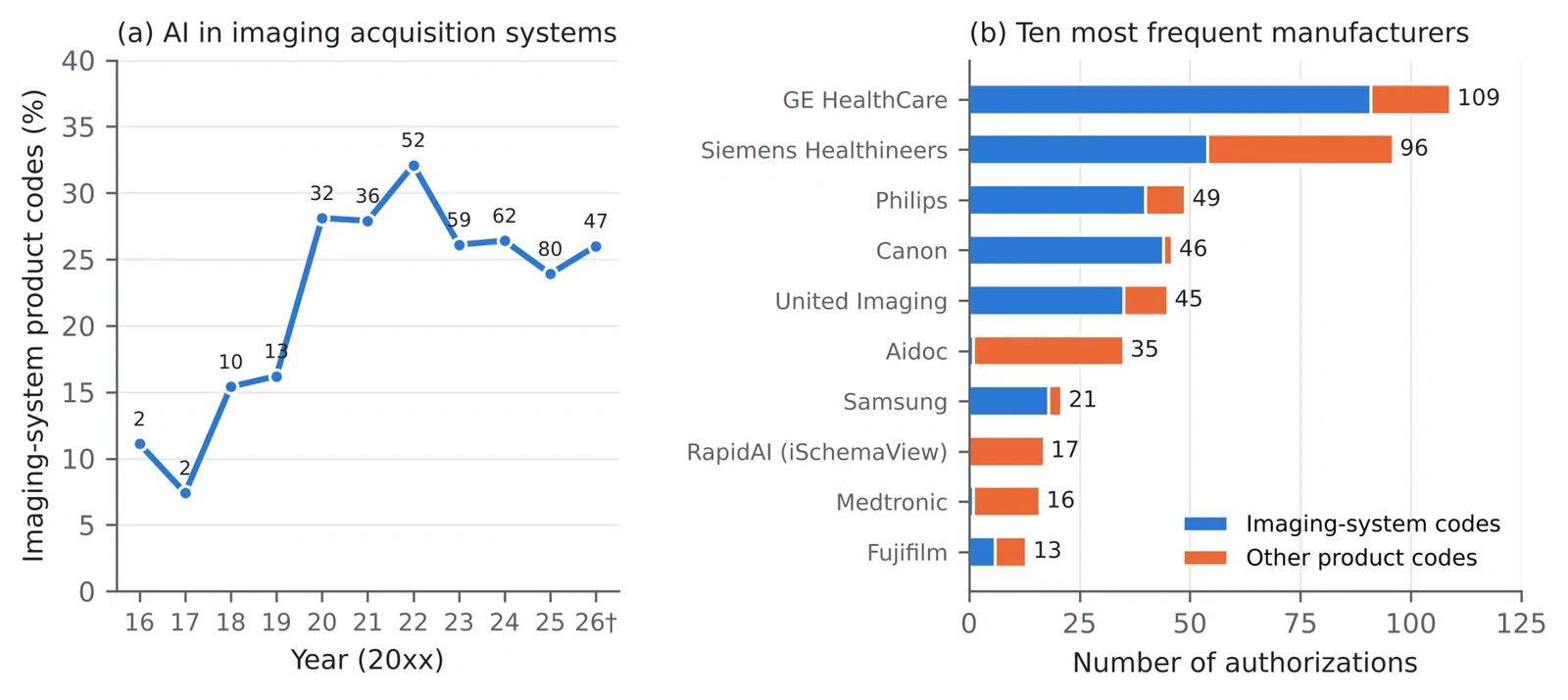
Artificial intelligence (AI) in imaging systems and the 10 most frequent manufacturers. (a) Share of annual authorizations made under imaging system product codes (ultrasound, computed tomography, magnetic resonance, emission tomography, and X-ray systems), 2016-2026; point labels show the number of imaging system entries in each year, and the 2026 point (marked with a dagger) covers January to June only. (b) The 10 manufacturer groups with the most entries, divided into imaging system product codes and other product codes.

Manufacturers

The manufacturer base was large and fragmented. Of the 559 manufacturer groups, 325 (58.1%) held a single listing, while 69 groups with five or more listings together accounted for 868 entries (53.8%). The 10 most frequent groups accounted for 447 entries (27.7%) (Figure 4): GE HealthCare (109), Siemens Healthineers (96), Philips (49), Canon (46), United Imaging (45), Aidoc (35), Samsung (21), RapidAI (17), Medtronic (16), and Fujifilm (13). The HHI was 133.5, which indicates a low level of concentration. Across all product codes, the seven major imaging manufacturers held 18 of 190 entries (9.5%) in 2016-2019, 123 of 405 (30.4%) in 2020-2022, and 236 of 977 (24.2%) in 2023-2026. In 2025, 87 manufacturer groups received their first listed authorization, and 277 of the 559 groups (49.6%) first appeared on the list in 2023 or later. Only two device names explicitly mentioned a predetermined change control plan.

Because successive clearances of the same product appear as separate entries, manufacturer counts partly reflect repeated listings. The 1,614 entries corresponded to 1,195 unique devices (1.35 entries per device), and the 419 repeat entries came from 144 manufacturer groups. Repeated listings were most common among the largest firms; for example, the 35 Aidoc entries corresponded to six product names, mostly successive clearances of its triage software for different findings. On a unique-device basis, the 10 most frequent groups held 288 of 1,195 devices (24.1%), compared with 27.7% of entries; the seven major imaging manufacturers held 249 (20.8%), compared with 23.5% of entries; 386 of 559 groups (69.1%) held a single device; and the HHI was 108.6. Concentration measures were therefore somewhat lower for unique devices than for entries, but the picture of a large, fragmented manufacturer base was unchanged.

Sensitivity analyses

After deduplication to 1,195 unique devices, radiology accounted for 878 (73.5%), the 510(k) pathway for 95.1%, and imaging system codes for 270 (22.6%). The radiology share followed the same pattern across periods (34.1%, 61.1%, 83.4%, and 74.3%), and radiology devices remained less likely to use a novel pathway (17 of 878 vs. 42 of 317; OR 0.13, p < 0.001). Restricting the analysis to entries through December 2025 (n = 1,433), the radiology share in 2023-2025 was 615 of 796 (77.3%), lower than in 2020-2022 (χ² = 11.93, p < 0.001), and the year-adjusted OR for a novel pathway was 0.15, 95% CI 0.08-0.27.

## Discussion

This study analyzed the full FDA AI-Enabled Medical Device List through June 2026, more than twice as many entries as the most recent analysis based on the 2023 list [2] and six months more than two analyses that ended in December 2025 [11,12]. Four main findings emerge. First, growth has not slowed: annual authorizations have increased by roughly a third per year for a decade, and 2025 was the largest year on record. Second, radiology remains dominant and still growing, but its share has fallen back from its 2020-2022 peak while the range of specialties has widened. Third, the regulatory route is overwhelmingly 510(k), radiology entries remain less likely than others to use novel pathways after accounting for era, and a small number of De Novo grants have generated product codes that now carry a large share of subsequent clearances. Fourth, about one in four entries concerns an imaging system rather than standalone software, and a few large manufacturers dominate this segment. All of these findings describe regulatory authorizations, not clinical adoption, use, or outcomes.

Comparison with recent analyses

The findings agree closely with the two recent analyses of the same registry. Using the same era boundaries, Lee and Youn reported a radiology share of 85.5% in 2020-2022 and 77.5% in 2023-2025 [11], compared with 85.7% and 77.3% for the corresponding entries here, and Golshani and Joseph reported that radiology accounted for 76.5% of authorizations through 2025 [12]. Beyond extending the observation period, the present study adds four elements: an era-adjusted analysis of regulatory pathway by specialty, the identification of De Novo product codes that were later reused by 510(k) clearances, a breakdown of product code concentration, and an analysis of AI embedded in imaging acquisition systems.

Growth and its interpretation

The growth rate observed here is consistent with earlier reports documenting rapid increases up to 2021, 2023, and 2025 [2,7,10,11], and it was robust to the choice of starting year and model. Analyses based on older versions of the list therefore cover a minority of the entries now on it. The 2026 figures should be interpreted with caution, because the FDA states that devices whose decision summaries had not yet been published during a data collection period are added in later updates [4]. Recent months are therefore likely to be undercounted, and the 2026 figures are best treated as provisional.

Specialty concentration and diversification

Radiology has dominated AI device authorizations since at least 2016, a finding shared by previous analyses [1,2,7,10-12]. Commonly proposed explanations include the abundance of standardized digital imaging data and the suitability of image interpretation tasks for deep learning [11,12], together with early classifications that created regulatory templates for detection, triage, and image processing software [8]. The present data show a reversal from the 2020-2022 peak rather than a sustained decline: the radiology share in 2023-2026 was similar to its pooled level before 2023, and radiology authorizations continued to grow in absolute terms. Diversification has therefore occurred alongside continued growth in radiology. In the registry, growth outside radiology came mainly from surgical navigation and EEG and sleep analysis in neurology, home sleep apnea testing in anesthesiology, computer-aided polyp detection in gastroenterology, and ECG analysis in cardiology. These categories are consistent with developments reported in the literature, such as randomized evidence for computer-aided polyp detection [19], but the registry cannot establish why these specialties grew, and part of the change may reflect how the FDA identifies AI-enabled devices [4]. Whether AI-enabled devices are increasingly used in these specialties is a separate question that authorization data cannot answer [11].

Regulatory pathways and predicate chains

Of the 1,614 entries, 1,553 (96.2%) were cleared through 510(k), and the share authorized through De Novo or PMA has declined over time. This is not, by itself, a sign of weak oversight, because 510(k) is the standard route for moderate-risk devices across the device sector [5]. However, it means that most AI devices reach the market on the basis of substantial equivalence to a predicate rather than independent evidence of clinical benefit. Muehlematter et al. showed that a substantial proportion of AI devices cleared in 2019-2021 had predicate networks that originated in devices without AI functionality [8], Wu et al. found that most evaluations were retrospective [9], and Windecker et al. reported clinical performance studies for 505 of 903 devices (55.9%) and recalls for 43 (4.8%) [20]. The present analysis complements these findings at the product code level: 22 of 40 De Novo grants established codes that were later used by 181 510(k) clearances, and a single 2018 stroke triage classification underpins 85 later clearances. De Novo special controls may therefore have a long reach, because they shape the evidence expected from later devices in the same code. Whether special controls for heavily reused AI product codes keep pace with the technology is a question for further regulatory research, because this study did not examine decision summaries or changes in predicate quality.

The lower use of novel pathways in radiology persisted after adjustment for period and calendar year, and in the years after 2012, when direct De Novo requests became available [5], so it does not appear to be explained by the changing specialty mix over time. A plausible structural explanation is cumulative access to predicates. Radiology accumulated authorized AI devices early, with the first radiology entry on the list, a mammography computer-aided detection system, authorized in 1998, which gave later devices a large pool of potential predicates, whereas novel devices in other specialties more often lack a suitable predicate. If predicate availability lies on the path between specialty and pathway, adjusting for it would remove part of the effect of interest rather than a confounder, so the OR is best read as describing the regulatory context of each specialty rather than an intrinsic characteristic of radiology or its propensity for innovation. Device risk class, which largely determines whether PMA is required, is not recorded in the list and could not be included in the models.

AI embedded in imaging systems

Nearly a quarter of entries (399 of 1,614, 24.7%) are regulated under classification codes for ultrasound, CT, MR, nuclear medicine, and X-ray systems. In these devices, AI typically supports image reconstruction, noise reduction, automated positioning, protocol selection, or measurement within the scanner, rather than producing a separate diagnostic output. This segment grew sharply around 2020 and has since remained at about a quarter of authorizations, and it is concentrated among a small number of large manufacturers, which held 288 of these 399 listings (72.2%).

Regulatory classification depends on a software function's purpose rather than its location within hardware, and the present data do not show whether AI functions embedded in imaging systems are reviewed differently from standalone software; any difference in scrutiny is an untested hypothesis. Embedded AI may also be less visible to radiologists, sonographers, and clinical engineering teams than standalone software, but this too has not been studied directly. Post-market evidence for AI devices is limited more generally. In an analysis of 943 adverse event reports linked to 823 cleared AI/ML devices in the FDA Manufacturer and User Facility Device Experience (MAUDE) database, Babic et al. found that most reports were coded as malfunctions, that many reflected user error, and that key context such as event location was missing, which makes it difficult to attribute events to AI functions [21]. The dataset analyzed here contains no adverse event information, so these points are offered as context rather than findings. Clearer labeling of AI functions within conventional devices, as encouraged in the FDA draft lifecycle guidance [14], could support procurement decisions, acceptance testing, and incident investigation in hospitals.

Manufacturer structure and lifecycle oversight

The manufacturer base is broad and continues to attract new entrants: 325 of 559 manufacturer groups (58.1%) have a single listed authorization, and 277 (49.6%) first appeared on the list from 2023 onward. A low HHI indicates an overall competitive field, but the picture differs by segment, with imaging system AI dominated by established equipment makers and standalone software spread across many smaller firms. These figures describe authorization activity, not the maturity of quality systems, and entry-based counts give more weight to firms that clear successive versions of the same product; concentration was somewhat lower when unique devices were counted. All manufacturers are subject to the same quality system requirements regardless of size, now aligned with ISO 13485 under the Quality Management System Regulation that took effect in February 2026 [22], and to the lifecycle expectations described in FDA guidance on AI-enabled devices [13-15]. Whether post-market surveillance capacity differs by firm size is an open question that the list, which contains no information on firm size, certification, or recalls, cannot address. FDA guidance on predetermined change control plans now provides a mechanism for planned modifications [13], but device names alone gave little indication of its uptake, and decision summaries would be needed to assess it.

Relevance for other regulators and health systems

Many regulators, including the SFDA, have issued AI-specific guidance [16,17], and the European Union (EU) has adopted a horizontal AI regulation that applies alongside its medical device regulations [18]. Because the FDA list is widely used as an international reference, the findings on growth and specialty concentration are relevant beyond the United States. The predominance of 510(k) clearances, however, reflects the US framework and should not be generalized to systems such as notified body assessment under the EU medical device regulations. Measures that follow from the literature and from this descriptive picture, offered as considerations rather than conclusions of the analysis, include public registries of authorized AI-enabled devices with structured fields for intended use, AI function, evidence type, and change control plans; explicit identification of AI functions embedded in conventional equipment at registration and procurement; and adverse event reporting that allows events to be linked to AI functions [20,21].

Limitations

This study has several limitations. First, the FDA list is not comprehensive. It is compiled mainly through AI-related terms in public summaries and classifications [4], it is updated irregularly, and devices can be added retrospectively when decision summaries are published, so the 1,614 entries are a snapshot of the list as accessed on October 15, 2026, and the identification method may have changed over time. Second, the analysis counts authorizations rather than unique devices: successive clearances of the same product appear as separate entries, which inflates the counts and concentration measures of firms that clear many versions, although the main findings were similar for 1,195 deduplicated devices. Repeated and related listings from the same manufacturer may also be correlated; this was handled with manufacturer-clustered standard errors and the deduplicated analysis, but residual dependence cannot be excluded, and the deduplication itself relied on device names and is approximate. Third, the lead review panel is an administrative category and does not necessarily indicate where a device is used clinically. Fourth, the classification of imaging systems was based on product codes; some entries under these codes, such as software accessories regulated under ultrasound codes, are not scanners, so this category is an approximation. Fifth, company name standardization relied on a rule list and may have missed some corporate relationships, such as acquisitions. Sixth, the final period includes only six months of 2026 and is affected by reporting lag, so its composition may change as delayed entries are added, although results were similar when the analysis ended in December 2025. Seventh, the design is cross-sectional, and the period comparisons are descriptive; a decline from a peak within a non-monotonic trajectory does not by itself establish sustained diversification, and the pathway-specialty association, although robust to adjustment for era, may reflect predicate availability and device risk class, which were not observed. Finally, the list contains six administrative fields and does not measure clinical use or outcomes, and it holds no information on clinical performance, validation, recalls, or adverse events. The analysis therefore cannot establish device safety or effectiveness, clinician awareness of AI functions, or the maturity of manufacturer quality systems. Linking the list to decision summaries, recall records, and adverse event data is a natural next step.

## Conclusions

FDA authorizations of AI-enabled medical devices have grown rapidly over the past decade, and most entries on the current list were authorized from 2023 onward. Radiology remains the dominant specialty and continues to grow in absolute terms, while other specialties are expanding alongside it. The 510(k) pathway accounts for almost all authorizations, radiology entries remain less likely than others to use novel pathways after accounting for era, and a small number of De Novo classifications underpin a large share of later clearances.

A substantial share of authorizations now concerns AI embedded in imaging equipment, mostly from a few large manufacturers. These findings describe regulatory authorizations rather than clinical adoption, performance, or safety. They point to questions for further study, including how AI functions in conventional equipment are labeled, how special controls for heavily reused product codes evolve, and how post-market surveillance can capture problems related to AI functions.

## Appendices

Appendix A: Imaging system product codes

Entries were flagged as imaging system entries when their primary product code was one of the following.

**Table 5:** Imaging system product codes.

Product code	Modality	Entries, n
IYN	Diagnostic ultrasound (pulsed Doppler imaging)	124
IYO	Diagnostic ultrasound (pulsed echo imaging)	12
JAK	Computed tomography	103
LNH	Magnetic resonance imaging	100
KPS	Emission computed tomography	26
OUO	Combined positron emission tomography and magnetic resonance	2
OWB	Interventional fluoroscopic X-ray	15
OXO	Image-intensified fluoroscopic X-ray	1
JAA	Fluoroscopic X-ray	4
KPR	Stationary radiographic X-ray	6
MQB	Solid-state X-ray imager	3
IZL	Mobile radiographic X-ray	1
OTE	Digital breast tomosynthesis	2
Total		399

Appendix B: Manufacturer standardization rules

Step 1. Each company name was converted to lowercase; text in parentheses and text after "doing business as" were removed; punctuation was replaced by spaces; and the legal suffixes inc, incorporated, ltd, limited, llc, co, corp, corporation, company, gmbh, ag, bv, nv, sas, sa, pty, plc, srl, spa, ab, oy, ehf, aps, as, uc, kk, lp, and pte were removed. Step 2. The resulting names were merged into the following groups when they matched the listed patterns; all other names were kept as their own group after Step 1.

**Table 6:** Manufacturer standardization rules table.

Manufacturer group	Source names merged into the group
GE HealthCare	Names beginning with "GE", GE Healthcare, GE Medical Systems, GE Hangwei, GE Hualun, General Electric
Siemens Healthineers	Any name containing Siemens
Philips	Any name containing Philips
Canon	Canon, Toshiba
Fujifilm	Any name containing Fujifilm
United Imaging	Any name containing United Imaging
Samsung	Any name containing Samsung
Medtronic	Medtronic, Medicrea
Hologic	Hologic
iCAD	iCAD
Riverain	Riverain
Viz.ai	Viz.ai
Aidoc	Aidoc
Qure.ai	Qure.ai
Annalise.ai	Annalise
Coreline Soft	Coreline
Infervision	Infervision
Cosmo Intelligent Medical Devices	Cosmo Artificial Intelligence
Iterative Health	Iterative Health, Iterative Scopes
Eko Health	Eko Devices, Eko Health
Us2.ai	Eko.ai doing business as Us2.ai
Imbio	Imbio
MIM Software	MIM Software (not MIM Fertility)
Perspectum	Perspectum
Zebra Medical Vision	Zebra Medical Vision
Esaote	Esaote
Dentsply Sirona	Dentsply Sirona
Stryker	Stryker
Abbott	Abbott
HeartFlow	HeartFlow
Arterys	Arterys
Avicenna.AI	Avicenna.AI
icometrix	icometrix
Subtle Medical	Subtle Medical
DeepHealth	DeepHealth
RapidAI	iSchemaView, RapidAI
Lunit	Lunit
Varian	Varian
Intuitive Surgical	Intuitive Surgical
